# Ideal number of target lesions per organ to measure in metastatic colorectal cancer

**DOI:** 10.3892/ol.2014.2409

**Published:** 2014-08-04

**Authors:** HYEONG SU KIM, JUNG HAN KIM

**Affiliations:** Department of Internal Medicine, Division of Hematology-Oncology, Kangnam Sacred-Heart Hospital, Hallym University Medical Center, Hallym University College of Medicine, Seoul 150-950, Republic of Korea

**Keywords:** target lesion, Response Evaluation Criteria in Solid Tumors 1.1, tumor response, colorectal cancer

## Abstract

The Response Evaluation Criteria in Solid Tumors (RECIST 1.1) guideline states that the two largest lesions per organ should be measured as target lesions for assessment of the tumor response. This criterion is considered to be arbitrary and, to the best of our knowledge, has not been supported by any objective evidence. The present study hypothesized that measuring the single largest lesion in each organ into which the cancer had metastasized (termed the modified RECIST; mRECIST 1.1) may yield the same response classification as measuring the two target lesions per organ (as per the RECIST 1.1 guideline). The medical records of patients with metastatic colorectal cancer (CRC), who received first-line chemotherapy between January 2004 and June 2013 were reviewed. The tumor responses of the patients were compared according to the two criteria using computed tomography. A total of 38 patients were included in the present study, all of whom had at least two target lesions in any one organ according to the RECIST 1.1 guidelines. When adopting the mRECIST 1.1, rather than the RECIST 1.1, 18 patients (47.4%) demonstrated an increase in the rate of change of the sum of the tumor measurements. The overall response rates of chemotherapy were 39.4% and 34.2% according to the RECIST 1.1 and the mRECIST 1.1, respectively, and the difference between the two criteria was not identified to be significantly different (P=0.226). The tumor response showed near perfect agreement between the RECIST 1.1 and mRECIST 1.1 criteria (κ=0.905). Only two patients (5.3%) showed a disagreement with regard to the tumor responses between the two criteria. Therefore, it was identified that the mRECIST 1.1 showed a high level of concordance with the original RECIST 1.1 guidelines in the tumor response assessment of metastatic CRC patients to chemotherapy. The present results indicate that the mRECIST 1.1, with a decreased number of target lesions to be measured, may be more convenient in clinical practice for the assessment of tumor response.

## Introduction

The accurate assessment of changes in tumor burden is critical for anticancer treatment as well as clinical trials of new drugs. Traditionally, tumor sizes have been measured bi-dimensionally by obtaining the product of the longest diameter and the longest perpendicular diameter of each tumor. In the early 1980s, the World Health Organization (WHO) developed the WHO response criteria in an attempt to standardize the methods for evaluating the tumor response (1). However, as the details for selecting and measuring the target lesions were not clearly described in the WHO guidelines, the assessment of the tumor response has not been accurately reproducible between studies (2). In clinical practice, measuring all of the target lesions using two dimensions and calculating the sums of their products may result in errors and is time-consuming.

In 2000, the Response Evaluation Criteria in Solid Tumors (RECIST) working group presented the RECIST guideline version 1.0, to clarify and simplify the tumor response criteria (3). Major features of the RECIST 1.0 guideline included the use of unidimensional measures, rather than the bi-dimensional measures previously recommended by WHO, for the evaluation of tumor size and instructions on the number of target lesions to be evaluated. The RECIST 1.0 recommended measuring a total of 10 target lesions, with a maximum of five per organ. Thus, the RECIST 1.0 guideline has been widely accepted as a standardized method for the assessment of the tumor response. However, a number of questions and issues regarding the number of target lesions, the size of lymph nodes (LNs) to be assessed and the utility of novel imaging technologies have been raised with regard to the RECIST 1.0 guidelines (4,5).

In 2009, the RECIST Working Group published the revised RECIST guideline version 1.1, based in part on the investigation of a database containing data from >6,500 patients from 16 clinical trials (5,6). The significant modifications to the RECIST 1.1 included updates concerning the maximum number of target lesions, the LN measurements and the definition of progressive disease (PD) (7,8). The maximum number of target lesions to be assessed was reduced from 10 to five in total, and from five to two target lesions per organ. While the total of 10 target lesions proposed in the RECIST 1.0 was an arbitrarily selected number, the RECIST 1.1 recommended the measurement of a total of five lesions, based on the analysis of patient data (6) and statistical simulation studies (9,10). However, the criterion of two target lesions per organ remains an arbitrary decision and, to the best of our knowledge, it has not been supported by any objective evidence (9). Zacharia *et al* (11) reported that measuring the single largest lesion showed approximately the same response classification as compared with measuring up to five target lesions in patients with liver metastases of colorectal cancer (CRC). This finding indicated that the ideal number of target lesions per organ required to accurately assess the tumor response remains to be determined in future studies.

The present study proposes the modified RECIST (mRECIST) 1.1, hypothesizing that measuring the single largest lesion in each organ into which the cancer had metastasized would yield approximately the same response classification as measuring the two target lesions per organ (as recommended by the RECIST 1.1 guidelines). In the present study, computed tomography (CT) was used to compare the tumor response assessment as obtained using the mRECIST 1.1 and the RECIST 1.1 guidelines in patients with metastatic CRC.

## Patients and methods

### Patients

The present study was performed under the institutional Review Board’s waiver (IRB no. 2014-02-20), according to the Korean Ethical Guidelines for Epidemiological Research. The medical records of patients with metastatic CRC were retrospectively reviewed. The selected patients had received either 5-flurouracil/leucovorin plus oxaliplatin (FOLFOX) or 5-flurouracil/leucovorin plus irinotecan (FOLFIR1) as a first-line chemotherapy treatment, between January 2004 and June 2013 at the Kangnam Sacred Heart Hospital (Seoul, South Korea). The chemotherapy regimens consisted of biweekly oxaliplatin (85 mg/m^2^ as a 90-min intravenous [i.v.] infusion on day one) or irinotecan (150 mg/m^2^ as a 2-h i.v. infusion on day one) plus 5-flurouracil/leucovorin (20 mg/m^2^ leucovorin as a bolus i.v. injection on day one, followed by 3,000 mg/m^2^ 5-flurouracil as a 46-h continuous i.v. infusion). Patients were considered to be eligible for inclusion in the present study according to the following criteria: i) A histologically identified adenocarcinoma of the colon or rectum; ii) a radiologically or histologically confirmed metastatic disease with at least two measurable lesions in any one organ according to the RECIST 1.1; iii) no history of another type of cancer; iv) no history of previous chemotherapy treatment except for adjuvant therapy; and v) CT tumor assessment at baseline and following chemotherapy.

### CT examinations

All CT examinations were performed using a 64-MDCT scanner (SOMATOM Sensation 64; Siemens AG Healthcare, Forchheim, Germany) with a slice thickness of 5 mm, following which, the scanned CT images were uploaded onto the Picture Archiving Communication System (PACS; PiView Star; INFINITT Healthcare Co. Ltd., Seoul, Korea). The CT scan images that were used for evaluating tumor response to chemotherapy were performed following four cycles of FOLFOX or FOLFIRI.

### CT tumor measurements

The tumor measurements of each patient were evaluated from the original CT images. CT tumor measurements were performed manually on axial CT image planes using the calipers of a measurement tool on the PACS. The target lesion description, CT size measurement, sum of the longest diameters of the target lesions, descriptions of any non-target lesions, and the best tumor response for each patient were recorded separately by two oncologists according to the RECIST 1.1 and mRECIST 1.1 guidelines. Briefly, measurements were taken of the short axis of the LN and LNs ≥15 mm were considered to be target lesions. LNs that measured ≥10 mm and <15 mm in the short axis were considered to be non-target lesions, and LNs with a short axis of <10 mm were regarded as normal. The maximum number of target lesions to be assessed was five, with a maximum of two per organ, according to the RECIST 1.1 guidelines or the single largest lesion in each organ according to the mRECIST 1.1. The diameter of each target lesion was defined as the mean of the values as measured by two separate oncologists. In cases where there was a discrepancy in the tumor measurements between the two medical oncologists, a board-certified abdomen radiologist re-evaluated the CT results.

### Definitions of tumor response

The definitions of treatment response, used throughout the present study, were in accordance with the original RECIST 1.1 guidelines. Complete response (CR) was defined as the complete disappearance of all tumor lesions. Partial response (PR) was defined as a reduction in the sum of tumor measurements by ≥30%. PD was defined as ≥20% increase in the sum of the tumor measurements. In addition, an absolute increase of ≥5 mm to the lesions was a prerequisite for PD. The appearance of new lesions or the substantial progression of non-target lesions was considered to be PD. All other forms of tumor response were classified as stable disease (SD).

### Statistical Analysis

A paired Student’s t-test was used to determine the statistical significance of changes in the number of target lesions at baseline between the RECIST 1.1 and mRECIST 1.1 guidelines. The χ^2^ test was used to compare the overall response rates (ORRs) between the two groups. All P-values were based on a two-sided hypothesis and P<0.05 was considered to indicate a statistically significant difference. The level of concordance of the tumor responses between the two criteria was assessed using kappa statistics; a κ value of >0.75 was interpreted as showing strong concordance.

## Results

### Patient characteristics

During the study period, a total of 82 patients with metastatic CRC received first-line chemotherapy with either the FOLFOX or FOLFIRI regimen. According to the RECIST 1.1, 18 patients (21.9%) had no target lesions and four had not been evaluated for tumor response, therefore, these patients were excluded from the study. According to the inclusion criteria, 22 patients (26.8%), who had only one target lesion in each organ that was exhibiting metastasis, were also excluded from the study. Thus, a total of 38 patients (46.3%), each of which had at least two measurable lesions in any one organ, were included in the final analyses.

The baseline characteristics of the patients are summarized in Table I. The patients consisted of 23 males (60.5%) and 15 females (39.5%) with a median age of 60 years (range, 42–78 years). A total of 32 patients (84.2%) had colon cancer, and the remaining six patients (15.8%) had rectal cancer. A total of 27 patients (71.1%) had well- or moderately differentiated adenocarcinoma and 11 (28.9%) had poorly differentiated adenocarcinoma. The most common metastatic site containing measurable target lesions was the liver (76.3%), followed by the LNs (34.2%) and the lungs (18.4%). According to the RECIST 1.1, 24 patients (63.1%) had target lesions in only one organ, which were most commonly observed in the liver. A total of 11 patients had target lesions in two organs, these were most commonly found in the liver and the LNs. There were only three patients who had target lesions in more than three organs. Of the 38 patients included in the study, 33 (86.8%) were treated with the FOLFOX chemotherapy regimen, and the remaining five (13.2%) received the FOLFIRI regimen.

### Number of target lesions

The number of target lesions according to the mRECIST 1.1 was significantly lower as compared with the number according to the RECIST 1.1 (p<0.0001). The median number of target lesions was two (range, 2–5) by the RECIST 1.1 and one (range, 1–3) by the mRECIST 1.1. When the mRECIST 1.1 was adopted, rather than the RECIST 1.1, no newly defined target lesions were identified in the metastatic sites of the patients.

### Tumor response

**Figure 1:**
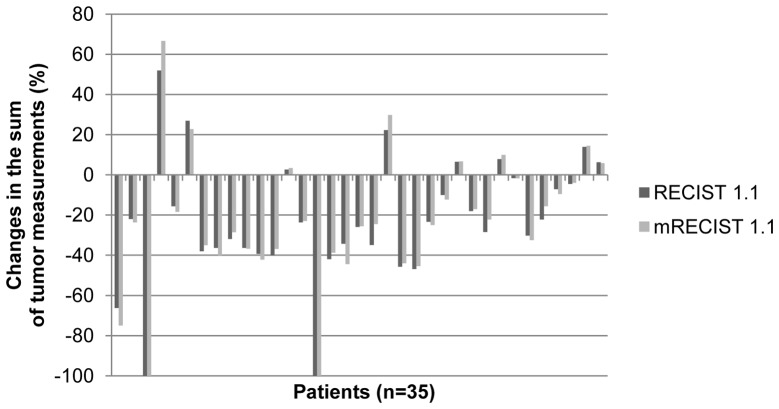
Changes in the sum of the tumor diameters of the target lesions according to the RECIST 1.1, as compared with the mRECIST 1.1. Three patients that were defined as having progressive disease, due to the presence of new lesions, are not included in the graph. RECIST 1.1, Response Evaluation Criteria in Solid Tumors 1.1; mRECIST 1.1, modified RECIST 1.1.

The changes in the sum of tumor measurements, according to the RECIST 1.1 and mRECIST 1.1 criteria, are presented as percentages in Fig. 1. Two patients demonstrated CR and three developed new metastatic lesions following chemotherapy. Among the remaining 33 patients, 18 (54.5%) showed an increase in the change rate (range, 0.1–14.7%) of the sum of the tumor measurements when adopting the mRECIST 1.1, rather than the RECIST 1.1. The remaining 15 patients (45.5%) demonstrated a reduction in the change rate (range, 0.4–10.5%) of the sum of the tumor measurements.

The comparison of the tumor responses between the RECIST 1.1 and mRECIST 1.1 guidelines is presented in Table II. No significant difference in the ORRs of FOLFOX or FOLIRI was identified between the two criteria; 39.5% (15/38) according to the RECIST 1.1 and 34.2% (13/38) according to the mRECIST 1.1 (P=0.226). Almost perfect agreement between the RECIST 1.1 and the mRECIST 1.1 was observed with regard to the tumor response assessment, with a κ value of 0.905 (95% confidence interval, 0.777–1.0). Two patients (5.3%) revealed a disagreement in the responses between the RECIST 1.1 and mRECIST 1.1 criteria. The two patients demonstrated PR according to the RECIST 1.1, however, were reclassified as SD according to the mRECIST 1.1.

## Discussion

The present study investigated the impact of measuring the single largest lesion in each organ with metastatic disease (termed the mRECIST 1.1), compared with measuring two target lesions per organ, as recommended by the RECIST 1.1, on the tumor response in patients with metastatic CRC. Single-lesion measurements significantly decreased the number of target lesions to be measured at the baseline of first-line chemotherapy. When compared with the two-lesion measurement, the single-lesion measurement had a concordant response classification in 94.7% of patients.

The WHO criteria and the RECIST guidelines depend on the changes in tumor size as determined using imaging techniques, therefore, target lesions are the most important radiological markers in the assessment of the tumor response. However, the criterion of a total of 10 target lesions, with a maximum of five lesions per organ, as proposed in the RECIST 1.0 guidelines was considered to be arbitrary and lacked objective evidence. Therefore, the RECIST Working Group retrospectively analyzed the effects of assessing one, two, three or five target lesions, as opposed to 10, on the tumor response and progression outcome using their patient database (6). It was observed that assessing three or five target lesions did not change the ORR or progression-free survival, when compared with assessing 10 lesions, as recommended by the RECIST 1.0 guidelines. A statistical simulation study for evaluating the impact of the number of target lesions also revealed little difference between five and 10 target lesions in the assessment of the overall tumor response (9). Based on these results, the RECIST 1.1 guideline proposed a total of five target lesions to be measured, with a maximum of two per organ.

Investigators have begun to use the RECIST 1.1 in clinical trials and in clinical practice anticipating that it will improve feasibility, as it is a more convenient assessment of tumor response (12). Almost perfect agreement between the RECIST 1.1 and the RECIST 1.0 has previously been demonstrated in the assessment of the tumor response in patients with NSCLC (12,13), advanced gastric cancer (14,15) and metastatic CRC (16). However, the criterion of assessing two target lesions per organ as per the RECIST 1.1 is considered to be an arbitrary value and, to the best of our knowledge, has not been supported by any objective evidence (9). Furthermore, in cases with more than three metastatic sites, the RECIST 1.1 may not be representative of all of the involved organs, due to the limited number of target lesions measured. The present study hypothesized that measuring the maximal diameter of the single largest lesion in each organ would be more representative of all of the metastatic sites. In the present study, of the 38 patients who had two or more measurable lesions in any organ, 24 patients (63.2%) had target lesions in only one organ, with the most common site being the liver. Three patients (7.9%) showed target lesions in three or more organs. However, no patients were identified to exhibit newly defined target lesions in the metastatic sites according to the mRECIST 1.1 guidelines.

There have been few studies investigating the optimal number of target lesions to be measured in order to assess the tumor response. Schwartz *et al* (10) simulated >1.8 million possible combinations of lesions from unidimensional measurements using a complex computerized model. The results indicated that the variance of the response assessment was decreased by 90% if at least four lesions were measured rather than just one lesion. Darkeh *et al* (17) investigated the minimum number of target lesions to measure, in order to represent the total number of target lesions, according to the RECIST 1.0 guidelines. In patients with five or more target lesions, measuring between four and seven lesions did not lead to a discrepancy; furthermore, the number of discordant cases was shown to increase gradually from measuring three lesions (4/53) to measuring one target lesion (8/53). On the basis of these results, the assessment of at least four lesions was recommended, when more than four target lesions are present. However, these studies evaluated the minimum number of target lesions, rather than considering the optimal number of target lesions, to measure per organ.

The present study compared the tumor response assessment between the mRECIST 1.1 (measuring the single largest lesion in each organ with metastases) and the RECIST 1.1 (measuring two target lesions per organ) in patients with metastatic CRC. Patients received either a FOLFOX or FOLFIRI regimen as first-line chemotherapy in a clinical practice setting, with no confirmation of tumor response. CT scans were performed following four cycles of chemotherapy, which translated into intervals of 8–12 weeks. The ORRs of the first-line chemotherapy according to the RECIST 1.1 and the mRECIST 1.1 were 39.4% and 34.2%, respectively. A significant limitation of the present study was the low number of patients that were assessed, however, the tumor responses showed a high level of concordance between the two criteria (κ=0.905). Of the 38 patients that were assessed, only two patients (5.3%) showed disagreement with regard to the responses during comparison of the two criteria; these two patients showed a PR according to the RECIST 1.1 and were reclassified as SD according to the mRECIST 1.1. Traditionally, in clinical practice, patients who are classified as PR or SD remain on the same treatment regimen. Therefore, with discordance only being shown between PR and SD classifications, the present study determined that the clinical impact of the mRECIST 1.1 on altering therapeutic decisions appeared to be minimal.

Prior to the release of the RECIST 1.1, Zacharia *et al* (11) reported that, in the majority of CRC patients with hepatic metastases, measuring the single largest lesion showed the same response classification as measuring up to five target lesions. Measuring two or more (up to five) target lesions showed complete concordance in the evaluation of the best tumor response, and single-lesion measurements gave a concordant tumor response in 93.3% (28/30) of patients as compared with the multiple-lesion measurements (κ=0.88). The above-mentioned findings, which are consistent with the results from the present study, indicate that it may be possible to reduce the number of target lesions measured per organ in clinical practice.

In conclusion, the mRECIST 1.1 demonstrated a high level of concordance with the original RECIST 1.1 guidelines in the response assessment of patients with metastatic CRC. As the clinical impact on therapeutic decisions appeared to be minimal, the mRECIST 1.1, with the decreased number of target lesions to be measured, may be more convenient for future use in clinical practice.

## Figures and Tables

**Table I:** Characteristics of the 38 patients (median age, 60 years; range, 42–78 years).

	Patients	
	
Characteristic	n	%
Gender		
Male	23	60.5
Female	15	39.5
Site		
Colon	32	84.2
Rectum	6	15.8
Histology		
Well- to moderately differentiated	27	71.1
Poorly differentiated	11	28.9
Measurable metastatic lesions		
Liver	29	76.3
Lungs	7	18.4
Lymph nodes	13	34.2
Peritoneum	4	10.5
Pancreas	1	2.6
Chemotherapy regimen		
FOLFOX	33	86.8
FOLFIRI	5	13.2

FOLFOX, oxaliplatin plus 5-flurouracil/leucovorin; FOLFIRI, irinotecan plus 5-fluoruracil/leucovorin.

**Table II:** Tumor response assessment by the RECIST 1.1, as compared with the mRECIST 1.1.

	Response by mRECIST 1.1		
	
Rresponse by RECIST 1.1	CR+PR (n=13)	SD (n=19)	PD (n=6)
CR + PR (n=15)	13	2	0
SD (n=17)	0	17	0
PD (n=6)	0	0	6

CR, complete response; PR, partial response; SD, stable disease; PD, progressive disease; RECIST, Response Evaluation Criteria in Solid Tumors; mRECIST, modified RECIST.
